# Hot Water Compresses in Tetanus

**Published:** 1881-03

**Authors:** 


### HOT WATER COMPRESSES IN TETANUS.

Warm or hot baths in tetanus have frequently been found to give great relief; but in many circumstances it is practically impossible to give them. In view of this, in the treatment of tetanus and trismus, Dr. Spoerer has successfully employed hot water compresses. He dips a large enough piece of coarse flannel in water of a temperature which can just be borne by the hand (50° to 55° C.), and applies the compress to the occiput and along the spine.

